# Accurate Tumor Delineation *vs.* Rough Volume of Interest Analysis for ^18^F-FDG PET/CT Radiomics-Based Prognostic Modeling inNon-Small Cell Lung Cancer

**DOI:** 10.3389/fonc.2021.726865

**Published:** 2021-10-18

**Authors:** Shima Sepehri, Olena Tankyevych, Andrei Iantsen, Dimitris Visvikis, Mathieu Hatt, Catherine Cheze Le Rest

**Affiliations:** ^1^ LaTIM, INSERM, UMR 1101, Univ Brest, Brest, France; ^2^ University Hospital Poitiers, Nuclear Medicine Department, Poitiers, France

**Keywords:** segmentation, radiomics, non-small cell lung cancer, machine learning, prognosis

## Abstract

**Background:**

The aim of this work was to investigate the ability of building prognostic models in non-small cell lung cancer (NSCLC) using radiomic features from positron emission tomography and computed tomography with 2-deoxy-2-[fluorine-18]fluoro-d-glucose (^18^F-FDG PET/CT) images based on a “rough” volume of interest (VOI) containing the tumor instead of its accurate delineation, which is a significant time-consuming bottleneck of radiomics analyses.

**Methods:**

A cohort of 138 patients with stage II–III NSCLC treated with radiochemotherapy recruited retrospectively (*n* = 87) and prospectively (*n* = 51) was used. Two approaches were compared: firstly, the radiomic features were extracted from the delineated primary tumor volumes in both PET (using the automated fuzzy locally adaptive Bayesian, FLAB) and CT (using a semi-automated approach with 3D Slicer™) components. Both delineations were carried out within previously manually defined “rough” VOIs containing the tumor and the surrounding tissues, which were exploited for the second approach: the same features were extracted from this alternative VOI. Both sets for features were then combined with the clinical variables and processed through the same machine learning (ML) pipelines using the retrospectively recruited patients as the training set and the prospectively recruited patients as the testing set. Logistic regression (LR), random forest (RF), and support vector machine (SVM), as well as their consensus through averaging the output probabilities, were considered for feature selection and modeling for overall survival (OS) prediction as a binary classification (either median OS or 6 months OS). The resulting models were compared in terms of balanced accuracy, sensitivity, and specificity.

**Results:**

Overall, better performance was achieved using the features from delineated tumor volumes. This was observed consistently across ML algorithms and for the two clinical endpoints. However, the loss of performance was not significant, especially when a consensus of the three ML algorithms was considered (0.89 *vs*. 0.88 and 0.78 *vs*. 0.77).

**Conclusion:**

Our findings suggest that it is feasible to achieve similar levels of prognostic accuracy in radiomics-based modeling by relying on a faster and easier VOI definition, skipping a time-consuming tumor delineation step, thus facilitating automation of the whole radiomics workflow. The associated cost is a loss of performance in the resulting models, although this loss can be greatly mitigated when a consensus of several models is relied upon.

## 1 Introduction

Non-small cell lung cancer (NSCLC) benefited from several improvements in diagnosis, staging, and treatment, but remains a deadly disease as the first cause of cancer death for men and the second for women (1). On the one hand, significant differences in the outcomes of patients have been observed depending on the clinical stage; hence, physicians rely on that factor to select a therapeutic strategy (i.e., concomitant or sequential combination of surgery, chemotherapy, and radiotherapy) (2). On the other hand, among patients with a similar stage, especially for stages II and III, highly variable outcomes (i.e., response to therapy and survival) have been reported.

Several studies showed the usefulness and the value of positron emission tomography/computed tomography (PET/CT) image modality using 2-deoxy-2-[^18^F]fluoro-d-glucose (^18^F-FDG) radiotracer for NSCLC staging, treatment planning, and monitoring (3). The clinical relevance of some of the new response metrics, such as the metabolically active tumor volume (MATV) and total lesion glycolysis (TLG), are under investigation. Commonly, the response to treatment is predominantly measured using the maximum standardized uptake value (SUV_max_) obtained within a tumor. However, it has many shortcomings: firstly, SUV_max_ is not capable of characterizing all types of uptake changes and associated responses. It can only precisely measure those responses that occur when there is a global change in the tracer uptake, i.e., when the uptake changes in the tumor are spatially homogeneous. Since SUV_max_ only involves a single voxel, it cannot capture changes in the shape of the tumor or in its spatial uptake distribution properties.

In recent years, various handcrafted quantitative features, known today as radiomics, have been introduced and investigated for their potential to quantify the intensity, shape, and heterogeneity of tracer uptake within the tumor volume on PET/CT images (4, 5).

Because radiomic features are typically extracted from a previously delineated tumor volume, the impact of the segmentation step on the resulting intrinsic value of radiomics has been examined in several studies. The robustness of a subset of textural features used to quantify ^18^F-FDG PET uptake, depending on the segmentation technique was first investigated in esophageal cancer treated with radiochemotherapy (6). A later study (7) investigated the test–retest variability of radiomic features in a dataset of 11 NSCLC patients with repeated scans and the inter-observer delineation variability in a set of 23 patients. Later, the impact of reconstruction and delineation was studied using 11 NSCLC full-body ^18^F-FDG PET/CT scans in order to investigate the repeatability and the effects of the reconstruction methods and delineation (8). The repeatability of the radiomic features to explore sensitivity to image reconstruction, noise, and the delineation method was further considered by the same team (9). The impact of tumor segmentation on the robustness of the features (10), on the reproducibility and non-redundancy of the features (11), or on the resulting prognostic value (12) were also investigated recently. On the one hand, all these studies showed that the choice of segmentation techniques can lead to substantial variations for some radiomic features, but all investigated the impact within the context of using the most accurate tumor volume to extract features. On the other hand, several studies recently compared the use of features extracted from delineated tumors *versus* these extracted from specifically different (larger or smaller) volumes of interest (VOIs), i.e., not necessarily containing the entire tumor or limited to the tumor extent.

A first study in the context of cervical cancer and FDG PET imaging investigated the predictive value of features (volume and total lesion glycolysis) extracted from VOIs of varying sizes by considering various thresholds from 30% to 70% of the SUV_max_, determining a variability of performance in the resulting models (13). A second work compared different segmentation volumes in differentiating uterine sarcoma from leiomyoma with preoperative imaging (14). The study compared three volumes: the tumor only, the tumor and the surrounding tissues, and the entire uterus. The best models were obtained by relying on features from the entire uterus [area under the receiver operating characteristic curve (AUC) = 0.876)] compared to the two other smaller VOIs (0.830 and 0.853 for tumor only and for tumor and the surrounding tissues, respectively). A third study investigated the impact of segmentation margin on machine learning (ML)-based high-dimensional quantitative CT texture analysis in the context of differentiating between low- and high-grade renal cancer (15). Two VOIs were compared: contour-focused *vs.* margin shrinkage of 2 mm. Features from the VOI with margin shrinkage were more reproducible than those from contour-focused VOI (93.2% *vs.* 86.2%); however, models combining contour-focused-derived features had better performance (AUC = 0.865–0.984 *vs*. 0.745–0.887).

One advantage of using larger VOIs containing the tumor could be to alleviate the need for accuracy in defining the VOI, hence facilitating and accelerating the whole radiomics analysis. Indeed, the accurate delineation of the tumor is often considered a significant time-consuming bottleneck step of the radiomics workflow.

The aim of this work was thus to investigate the ability of building prognostic models in NSCLC using radiomic features from ^18^F-FDG PET/CT images based on a “rough” VOI containing the tumor volume instead of the accurately delineated tumor. We hypothesized that a combination of features extracted from this larger VOI may capture the relevant information in a different manner than those calculated in the delineated tumor volume and still enable prediction of the outcome, sparing the cost of the delineation step. In that context, it is expected that shape features might become less informative in the rough VOIs compared to those calculated on the delineated tumor and that additional and/or alternative intensity or textural features will be selected in the models instead.

## 2 Materials and Methods

### 2.1 Patient Cohort

Since stage 1 patients have a very different (and more favorable) prognosis compared to those with stage II or III disease, mostly driven by treatment [(surgery *vs*. (chemo)radiotherapy], we focused here on patients with stage 2 and 3 tumors, where the potential impact of radiomics is likely to be the most important (16).

The inclusion criteria were confirmed NSCLC, stage 2 or 3; curative (chemo)radiotherapy treatment, and pretreatment FDG PET/CT imaging. Data from 138 NSCLC patients treated at the University Hospital of Poitiers, France, were collected (**Table 1**). The data of the first 87 patients were collected retrospectively, whereas the next 51 patients were recruited prospectively within the PRINCE project (INCa, PRTK-2015, registered trial NCT03199599). The study was conducted according to the guidelines of the Declaration of Helsinki. Ethical review and approval were waived for this study because the data were already collected for routine patient management before analysis, in which patients provided informed consent. No additional data were specifically collected for the present study. The exact same cohort of patients was recently analyzed in another study focusing on the comparison and fusion of ML algorithms, so the present results are directly comparable with that previous work (17).

**Table 1:** Patient characteristics.

	Characteristics	No. of patients (*N* = 138)	Training/validation set (*N* = 87)	Test set (*N* = 51)
Gender	Male	106	62	44
	Female	32	25	7
Age (years)	Range	46–94	46–94	46–89
	Mean ± SD	71.43 ± 9.44	71.35 ± 9.37	71.55 ± 10.00
Treatment	Radiotherapy only	68	30	28
	Chemoradiotherapy	70	57	23
Histology	Adenocarcinoma	82	51	29
	Squamous cell carcinoma	56	36	22
Clinical stage	I	0	0	0
	II	43	26	17
	III	95	61	34
	IV	0	0	0

### 2.2 PET/CT Imaging

All patients underwent a combined ^18^F-FDG PET/CT acquisition as part of the diagnosis and staging before treatment. A Biograph mCT 40 ToF with axial field of view of 21.6 cm (Siemens, Erlangen, Germany) was used, relying on the routine clinical protocol. PET/CT acquisition began after 6 h of fasting and 60 ± 5 min after injection of 2.5 MBq/kg of ^18^F-FDG (421 ± 98 MBq, range = 220–695 MBq). Non-contrast-enhanced, non-respiratory-gated (free breathing) CT images were acquired (120 kVp; Care Dose^®^ current modulation system) with an in-plane resolution of 0.853 × 0.853 mm^2^ and a 5-mm slice thickness. PET data were acquired using 3.5 min per bed position, and images were reconstructed using a CT-based attenuation correction and the standard routine clinical protocol, as we recently showed no improvement in the prognostic value of radiomic features when using different settings (either smaller voxels or smaller full width at half maximum of the Gaussian post-filtering) (18): OSEM-TrueX-TOF algorithm, with time-of-flight and spatial resolution modeling (three iterations and 21 subsets, 5-mm 3D Gaussian post-filtering; voxel size, 4 × 4 × 4 mm^3^).

### 2.3 Radiomics Analysis

#### 2.3.1 Preprocessing

As the PET images were reconstructed on a matrix with isotropic voxels, no further image interpolation was performed. CT images were interpolated to isotropic 1 × 1 × 1 mm^3^ voxels using linear interpolation.

PET images were converted into SUV using patient weight. Low-dose CT images were processed in Hounsfield unit (HU).

#### 2.3.2. VOI Definition and Segmentation

**Figure 1:**
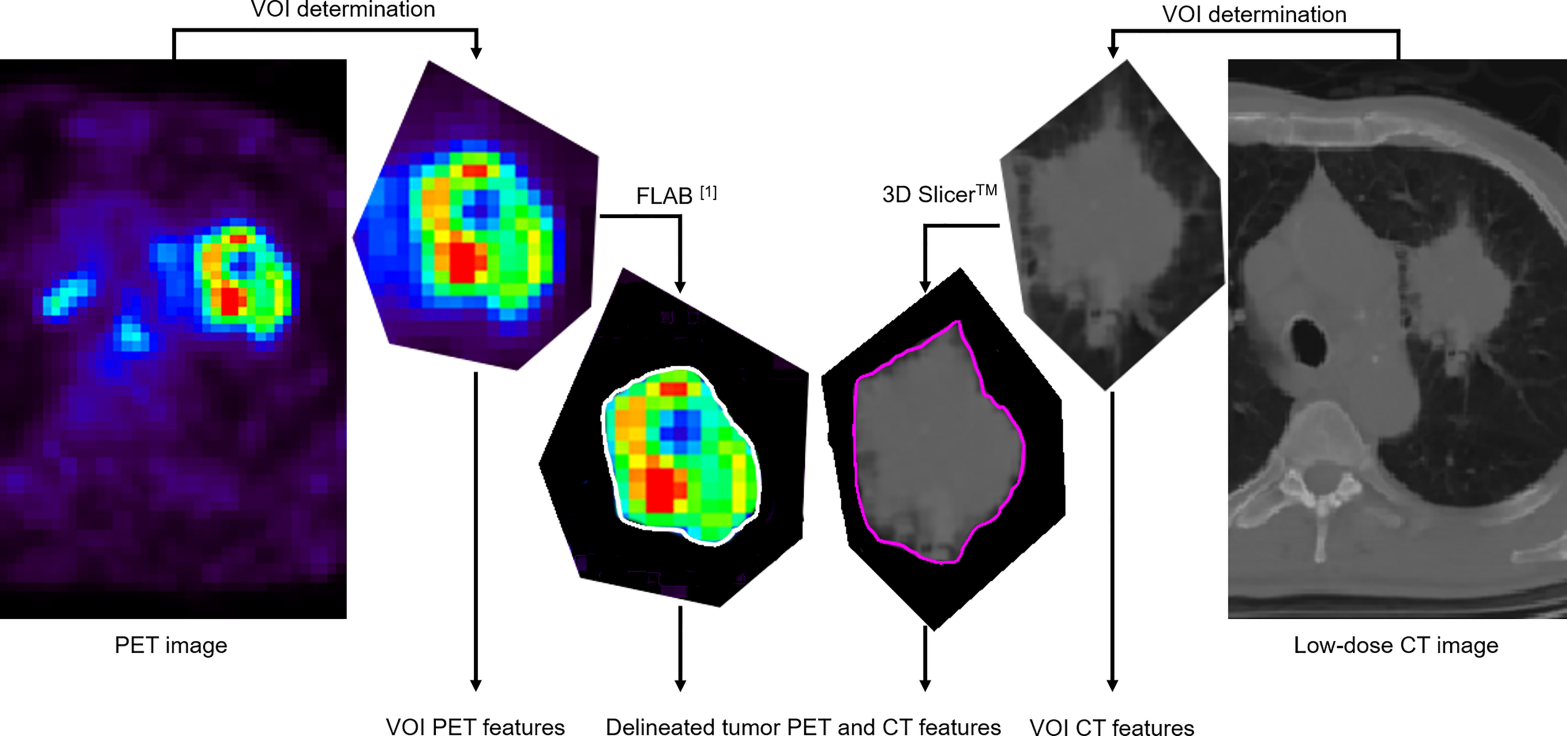
Both PET and low-dose CT images of the primary tumor are processed in the same manner: a volume of interest (VOI) containing the tumor is first manually determined. Radiomic features are extracted from this VOI (denoted “VOI features”). Then, segmentation of the tumor volume is carried out within the VOI with a (semi)automated algorithm. Radiomic features are then extracted from the delineated volume (this is the usual workflow).

Only the primary tumors were considered. PET and CT images were segmented independently by a single expert. The first step consisted of manually defining a “rough” VOI containing the tumor and its surroundings in both modalities. This is the usual first step in facilitating the automated or semi-automated tumor delineation by excluding the surrounding physiological uptakes or normal structures that should not be included in the tumor-only analysis. The tumor metabolic volume was then obtained in PET by applying the FLAB algorithm (19, 20) (MIRAS v1.0, LaTIM INSERM UMR 1101, Brest, France) in the manually defined “rough” VOI. The anatomical volume was obtained from the low-dose CT rough VOI semi-automatically by relying on the *Growcut effect* function of 3D Slicer™ (21). All delineations were checked and validated by an expert physician (C. Cheze Le Rest). **Figure 1** illustrates this process.

For the rest of the radiomics workflow, two different volumes for both PET and CT images were thus considered: the delineated tumor volume and the rough VOI.

#### 2.3.3 Radiomic Feature Extraction

Seventy-three radiomic features (14 shape, 10 intensity, and 49 textural) (see **Supplemental Table 1**) compliant with the most up-to-date imaging biomarker standardization initiative (IBSI) benchmark (22) were extracted using homemade software (MIRAS v1.0, LaTIM INSERM UMR 1101, Brest, France). Three different grey-level discretization methods [fixed bin number (FBN) with 64 bins, fixed bin width (FBW) with 0.5 SUV or 10 HU, and histogram equalization with 64 bins] were considered for second- and higher-order textural features. Note that the FBN and FBW discretization schemes are IBSI-compliant, but the histogram equalization, although mentioned by the IBSI, is not yet a standard. Texture matrices were implemented in 3D following the merging strategy (i.e., considering all 13 directions simultaneously). More details on the entire radiomics workflow are provided in **Supplemental Table 2**. A total of 147 features (10 + 14 + 49 × 3) were thus extracted from each tumor volume in both PET and CT, leading to 294 image-derived variables for each patient. These 294 features were extracted from both the tumor delineated volumes and the rough VOI.

#### 2.3.4 Modeling

All available clinical variables (age, gender, stage, treatment, and histology) and the PET and CT radiomic features were grouped into a single set to be processed by each of the ML pipelines. The two different sets corresponding to the two approaches (delineated tumor *vs*. rough VOI) were processed independently using the exact same data split and ML pipeline for a fair comparison.

Data were split into a training/validation set (*n* = 87 retrospectively recruited patients, 63%) and a test set (*n* = 51 prospectively recruited patients, 37%) (**Table 1**).

The classification task was set as a binary identification of patients with overall survival (OS) below 6 months (unbalanced, *n* = 15 in the training set and *n* = 9 in the test set) or below the median OS (balanced). In the case of the 6-month prediction, the synthetic minority oversampling technique (SMOTE) was implemented to facilitate the training of models.

The ML pipelines consisted of three algorithms with embedded feature selection and a consensus: support vector machine (SVM) with recursive feature elimination (RFE), random forest (RF) with embedded wrapper (EW), and logistic regression (LR) with features selected using least absolute shrinkage and selection operator (LASSO). Hyperparameters of the algorithms (e.g., the number of trees in RF) were optimized through five-fold cross-validation in the training/validation set. To generate a consensus model, the output probabilities of each of the three algorithms were averaged and binarized (> or ≤0.5), as this approach provided better results than did majority voting in our previous work (17).

The performance of the models in the training/validation set was assessed using accuracy, balanced accuracy (BAcc) in the case of the 6-month OS prediction, and the combination of sensitivity (Se) and specificity (Sp), favoring models with higher Sp and with a smaller number of features (lower complexity and higher potential for generalizability) for similar levels of BAcc. For models with similar overall accuracy values, a higher specificity is more clinically relevant, as patients who would be falsely identified as having poor prognosis might be offered palliative (or intensified) treatment when the standard treatment would actually benefit them. Importantly, none of the data from the test set were used in the training and optimization step of any of the models under comparison (either each of the models or the ensemble through averaging). All models were first finalized and optimized in the training set before final evaluation without any further modifications in the test set.

The best models obtained through each ML algorithm in training/validation set were then applied to the test set for final evaluation and to allow for relevant comparisons (i.e., models trained using input features from the accurately delineated tumor *vs*. those from the “rough” VOI). In order to provide some reference comparison, we also determined the accuracy reached by using only the clinical features as input to the ML pipelines or by relying only on clinical staging (stage 2 *vs*. 3), as previously reported (17).

Finally, although the present work focuses on the question of the input VOI for the performance of the models rather than the actual development of a prognostic model, we nonetheless auto-evaluated our study using the radiomics quality score (RQS) (23).

## 3 Results

Our study scored moderately on the RQS (see **Supplemental Table 3**) at 16 (19 when the data will be made available) out of 36, which is nonetheless higher than that of the average of studies reported recently (23–25).

The average follow-up was 41 months, with a minimum of 1.1 months and a maximum of 95 months. Median OS was 14.4 months, ranging between 1.1 and 50 months.

All results from the different models and the two outcome prediction tasks are presented for the training and test sets in **Table 2**.

**Table 2:** Performance comparison of the ML techniques using either features from the delineated tumor (D) or from the rough VOI (V), in addition to the available clinical factors.

ML	Task	VOI^a^	Training set			No. of features	Test set		
			Se	Sp	BAcc		Se	Sp	BAcc
LR	Median OS	D	0.67	0.77	0.72	37	0.54	0.75	0.63
		V	0.58	0.68	0.63	24	0.59	0.57	0.58
	6-month OS	D	0.81	0.87	0.84	45	0.8	0.76	0.78
		V	0.74	0.78	0.76	32	0.61	0.65	0.63
RF	Median OS	D	0.87	0.91	0.89	25	0.60	0.75	0.67
		V	0.75	0.86	0.87	23	0,53	0.59	0.56
	6-month OS	D	1	1	1	47	0.74	0.86	0.80
		V	0.83	0.89	0.86	58	0.73	0.75	0.74
SVM	Median OS	D	1	1	1	27	0.53	0.73	0.64
		V	0.82	0.82	0.82	20	0.56	0.60	0.58
	6-month OS	D	0.88	0.96	0.92	38	0.76	0.74	0.75
		V	0.84	0.90	0.87	43	0.75	0.77	0.76
Fusion (average of output probabilities)	Median OS	D	1	1	1	-	0.76	0.80	0.78
		V	0.93	0.89	0.90	-	0.76	0.78	0.77
	6-month OS	D	1	1	1	-	0.91	0.87	0.89
		V	0.88	0.94	0.91	-	0.98	0.78	0.88

ML, machine learning; VOI, volume of interest; Se, sensitivity; Sp, specificity; BAcc, balanced accuracy; LR, logistic regression; RF, random forest; SVM, support vector machine.

^a^D stands for the accurately delineated tumor and V for the “rough” VOI.

Models trained using only the clinical variables as input did not significantly improve the performance over clinical stage alone (BAcc <0.60 for all ML pipelines and both endpoints in the training set and <0.55 in the test set).

Overall, the level of accuracy achieved by the models relying on radiomic features was superior to that of clinical stage alone (BAcc values of 0.58 and 0.53 using stage 2 *vs*. stage 3 classification, respectively, as previously reported) (17), and the models were better at predicting very poor prognosis (6-month OS endpoint) than median OS. Some of the radiomics models included one or two clinical variables (staging and/or treatment), but mostly relied on the histogram, shape (except for the models trained using rough VOI features), and textural features. The drop of performance between the training/validation and test sets also suggests some overfitting.

Regarding the question addressed in this work, it was observed that the radiomic features extracted from the delineated primary tumor volume were slightly more informative than those extracted from a rough VOI. Indeed, models built with the three ML pipelines combining rough VOI features obtained a slightly lower performance (BAcc values of 0.57 ± 0.01 and 0.71 ± 0.07 for median OS and 6-month OS, respectively) than those exploiting delineated tumor features (BAcc values of 0.65 ± 0.02 and 0.78 ± 0.03). In both cases, the differences were not significant at the *p* < 0.01 or 0.05 levels (*p* = 0.059 for median OS and *p* = 0.286 for 6-month OS).

These models, however, relied on a similar number of features, i.e., models using rough VOI features did not need a larger number of features. Notably, shape features were not included in the models based on the rough VOI, contrary to those relying on the delineated tumor.

These observations were consistent for both endpoints (median OS and 6-month OS).

However, when looking at the consensus models (fusion of the output probabilities of each of the three pipelines, as the average of the outputs), the advantage of relying on the delineated tumor rather than on the rough VOI was greatly reduced, the two showing almost the same performances, with improved predictive ability compared to each independent ML algorithm, as previously reported (17): 0.89 *vs.* 0.88 for the 6-month OS endpoint and 0.78 *vs.* 0.77 for median OS, respectively.

## 4 Discussion

The main finding of our work is that, although radiomic features extracted from delineated tumor seemed more informative than those extracted from a simple rough VOI containing the tumor, almost as good results can be achieved without the need for the tedious and time-consuming (semi)automated delineation in the radiomics workflow, especially in the context of relying on a consensus of several ML techniques (in that case, the performance was almost equal). This could imply consequences regarding the way radiomics analyses are carried out since avoiding the need for actual tumor delineation before feature extraction could simplify and facilitate the whole process, at a very small cost in the resulting performance of the built models.

As expected, no shape features were used by the models to predict outcomes when features were extracted from the rough VOI, contrary to when then are extracted from the delineated tumor. Although there is an obvious correlation between the size and shape of the VOI and that of the tumor (larger, more complex tumors require larger and more complex VOIs to encompass them), there are most likely fewer differences between the various VOIs shapes to allow for patient differentiation. These features were replaced by alternative intensity and/or textural metrics in the VOI models. Although some of the models retained clinical variables (only clinical staging and treatment being selected), relying only on clinical factors provided the models with only limited accuracy (<0.60 in training and <0.55 in testing), and only models incorporating radiomic features had good performance in the test set.

Our work has several limitations. Firstly, the cohort used was collected from a single center. It allowed us to focus on the question at hand without having to deal with harmonization issues (26, 27) since all patients had their PET/CT acquisition in the exact same PET/CT system, with no variability in the acquisition protocol or reconstruction settings. However, this means that our findings will need to be validated in external datasets, for which we will implement harmonization techniques for handling the multicenter nature of the data (28). Although our cohort included both retrospectively (for training) and prospectively (for testing) recruited patients, the size of the test set was small as we could not include all available patients because a minimum follow-up duration was not reached and the prospective recruitment is still ongoing. This limited the statistical power for comparing the different results obtained with or without tumor delineation. However, the observed trends were systematic across all ML techniques and their consensus, strengthening our confidence in the potential generalizability of our results. The VOI determination and the tumor delineations were carried out by a single expert using a single method, which prevented us to compare the scale of inter-observer (or inter-segmentation method) variability with the differences between the delineated tumor and VOI features. The sensitivity of the results with respect to (moderate and reasonable) changes in the size or shape of the rough VOI was also not explored in the present work. It is expected to be obviously lower than the differences observed between the results obtained when exploiting features either from the rough VOI or from the accurately delineated tumor, which are already small. Finally, in order to fully automate the process for facilitating the radiomics workflow, the “rough” VOIs, which were manually created in the present work, should be reproduced by training a deep convolutional neural network (CNN) such as the U-Net, in a similar fashion, as we have recently demonstrated the feasibility regarding accurate tumor delineation (29). This way, the “rough” VOI could be obtained in a fully automated manner from the input PET/CT images without the need for a user intervention.

Several expansions of this work will be considered, such as a thorough comparison with deep learning-based feature extraction (“deep features”) and a validation of our findings in our extended prospective cohort: about 150 patients prospectively recruited in the PRINCE project should be available for this analysis once the follow-up duration of at least 1 year will be reached for all patients. Further validation of these findings will also be carried out in external datasets.

## Data Availability Statement

The original contributions presented in the study are included in the article/**Supplementary Materials**, further inquiries can be directed to the corresponding author/s.

## Ethics Statement

Ethical review and approval was not required for the study on human participants in accordance with the local legislation and institutional requirements. Indeed the patients/participants provided their written informed consent that their data could be collected for future research purposes as part of their routine management. No additional data was specifically collected for the present study.

## Author Contributions

CC, MH, and DV conceptualized the study. SS, OT, AI, and MH helped with the methodology. SS helped with software. SS and OT contributed to the formal analysis. SS, OT, and CC curated the data. SS and MH prepared the original draft. SS, OT, AI, CC, DV, and MH reviewed and edited the paper. CC and MH helped with funding acquisition. All authors contributed to the article and approved the submitted version.

## Funding

This work was funded by the French Ministry of Research (PhD grant), the INCa and DGOS through a grant in the PRINCE project (PRTK-2015, #R16063NN), the PREDICT Innovative Training Network funded by the Marie Sklodowska-Curie Actions, part of the EU’s Horizon 2020 Programme (grant agreement no. 766276), and the POPEYE project under the frame of ANR (Agence National de la Recherche; ANR-19-PERM-0007 POPEYE) and of ERA PerMed (POPEYE T11EPA4-00055).

## Conflict of Interest

The authors declare that the research was conducted in the absence of any commercial or financial relationships that could be construed as a potential conflict of interest.

## Publisher’s Note

All claims expressed in this article are solely those of the authors and do not necessarily represent those of their affiliated organizations, or those of the publisher, the editors and the reviewers. Any product that may be evaluated in this article, or claim that may be made by its manufacturer, is not guaranteed or endorsed by the publisher.
