# Supplementary material for: Accurate Tumor Delineation vs. Rough Volume of Interest Analysis for 18F-FDG PET/CT Radiomics-Based Prognostic Modeling inNon-Small Cell Lung Cancer
Source: Front Oncol. 2021 Oct 18;11:726865. doi: 10.3389/fonc.2021.726865 (PMC8560021; doi:10.3389/fonc.2021.726865)
Supplement: Supplementary file 1 [file DataSheet_1.docx]

Supplementary Material

# Supplemental table 1: list of features

| HISTOGRAM | Min_hist |
| --- | --- |
|  | Max_hist |
|  | Mean_hist |
|  | Variance_hist |
|  | Standard_Deviation_hist |
|  | Skewness_hist |
|  | Kurtosis_hist |
|  | Energy_hist |
|  | Entropy_hist |
|  | AUC_hist |
| SHAPE | Volume |
|  | 3D surface |
|  | ratio 3ds_vol |
|  | Compactness_v1 |
|  | Compactness_v2 |
|  | Spherical_disproportion |
|  | Sphericity |
|  | Asphericity |
|  | Max_3D_diam |
|  | Major_axis_length |
|  | Minor_axis_length |
|  | Least_axis_length |
|  | Elongation |
|  | Flatness |
| GLCM | Max_cooc |
|  | Average_cooc |
|  | Variance_cooc |
|  | Entropy_cooc |
|  | DAVE_cooc |
|  | DVAR_cooc |
|  | DENT_cooc |
|  | SAVE_cooc |
|  | SVAR_cooc |
|  | SENT_cooc |
|  | ASM_cooc |
|  | Contrast_cooc |
|  | Dissimilarity_cooc |
|  | Inv_diff_cooc |
|  | Inv_diff_norm_cooc |
|  | IDM_cooc |
|  | IDM_norm_cooc |
|  | Inv_var_cooc |
|  | Correlation_cooc |
|  | Autocorrelation |
|  | Tendency_cooc |
|  | Shade |
|  | Prominence_cooc |
|  | IC1 |
|  | IC2 |
| GLRLM | SRE_align |
|  | LRE_align |
|  | GLNU_align |
|  | RLNU_align |
|  | RP_align |
|  | LGSRE_align |
|  | HGSRE_align |
|  | LGHRE_align |
|  | HGLRE_align |
|  | RLNU_norm_align |
|  | RLVAR_align |
|  | Entropy_align |
| GLSZM | SZSE |
|  | LZSE |
|  | SZLGE |
|  | SZHGE |
|  | LZLGE |
|  | LZHGE |
|  | GLNU_area |
|  | ZSNU |
|  | ZSP |
|  | ZSNU_norm |
|  | ZSVAR |
|  | Entropy_area |

# Supplemental table 2: details of overall radiomics workflow

| **GENERAL** |  |
| --- | --- |
| Image acquisition | A Biograph mCT 40 ToF with axial field of view of 21.6 cm (Siemens, Erlangen, Germany) was used. PET/CT acquisition began after 6 hours of fasting and 60±5 min after injection of 2.5 MBq/kg of ^18^F-FDG (421±98 MBq, range 220-695 MBq). Non-contrast enhanced, non-respiratory gated (free breathing) CT images were acquired (120 kVp, Care Dose® current modulation system) with an in-plane resolution of 0.853×0.853 mm^2^ and a 5 mm slice thickness. PET data were acquired using 3.5 minutes per bed position and images were reconstructed using a CT-based attenuation correction and OSEM-TrueX-TOF algorithm (with time-of-flight and spatial resolution modeling, 3 iterations and 21 subsets, 5 mm 3D Gaussian post-filtering, voxel size 4×4×4 mm^3^). |
| Volumetric analysis | Imaging volumes were analyzed as fully-connected volumes (3D). |
| Workflow structure | PET and CT are both segmented individually (either rough VOI or tumor delineation), images are pre-processed (see below) and then features are extracted (see below). |
| Software | MIRAS v1.0, LaTIM, Brest, France. |
| **IMAGE PRE-PROCESSING** |  |
| Conversion | CT images were processed as HU and PET as SUV after standard conversion based on injected activity and weight. |
| Processing | No additional processing beyond interpolation and discretization (see below). |
| **ROI SEGMENTATION**^a;b^ | Only the primary tumors were considered. PET and interpolated CT images were segmented independently by a single expert. The first step consisted in manually defining a “rough” VOI containing the tumor and its surroundings in both modalities. The tumor metabolic volume was then obtained in PET by applying the FLAB algorithm implemented in MIRAS v1.0. The anatomical volume was obtained from the low dose CT rough VOI semi-automatically by relying on the Growcut effect function of 3D SlicerTM. All delineations were checked and validated by an expert physician (C. Cheze Le Rest) |
| **INTERPOLATION** |  |
| Voxel dimensions | PET: 4 × 4 × 4 mm^3^. CT: 0.83 × 0.83 × 5 mm^3^ – interpolated to 1 × 1 × 1 mm^3^. |
| Image interpolation method | No interpolation for PET, linear interpolation for CT. |
| Intensity rounding | N/A. |
| ROI interpolation method | N/A. |
| ROI partial volume | N/A. |
| **ROI RE-SEGMENTATION** |  |
| Inclusion/exclusion criteria | None. |
| **IMAGE DISCRETIZATION** |  |
| Discretization method | Fixed bin number (FBN), fixed bin width (FBW) and histogram equalization. |
| Discretization parameters | FBN: 64 bins, FBW: 0.5 SUV or 10 HU and histogram equalization: 64 bins |
| **FEATURE CALCULATION** |  |
| Features set | See supplemental table 1. |
| Features parameters | 26-voxel connectivity in 3D, 13 directions and texture matrices by merging. |
| **CALIBRATION** |  |
| Image processing steps | No deviation from IBSI. |
| Features calculation | All implemented features match the current benchmark of the IBSI. |

# Supplemental table 3: radiomics quality score

| **Item** | **Points** | **Current Study** | **Justification/remark** |
| --- | --- | --- | --- |
| Image protocol quality – well-documented image protocols (e.g.. contrast. slice thickness. energy. etc.) and/or usage of public image protocols allow reproducibility/replicability | +1 (if protocols are well-documented)+1 (if public protocol is used) | 2 |  |
| Multiple segmentations – possible actions are: segmentation by different physicians/algorithms/software. perturbing segmentations by (random) noise. segmentation at different breathing cycles. Analyze feature robustness to segmentation variabilities | +1 | 0 | This is a limitation of the study. Variability to multiple segmentation/experts should be carried out in the future |
| Phantom study on all scanners – detect inter-scanner differences and vendor-dependent features. Analyze feature robustness to these sources of variability | +1 | 0 | Not relevant to the present study, as the context is monocentric with a single scanner |
| Imaging at multiple time points – collect individuals’ images at additional time points. Analyze feature robustness to temporal variabilities (e.g.. organ movement. organ expansion/shrinkage). | +1 | 0 | Not relevant to the present study as the goal is prognosis at diagnosis/staging |
| Feature reduction or adjustment for multiple testing – decreases the risk of overfitting. Overfitting is inevitable if the number of features exceeds the number of samples. Consider feature robustness when selecting features | −3 (if neither measure is implemented)+3 (if either measure is implemented) | 3 | We use state-of-the-art robust embedded feature selection and combination through three different machine learning pipelines, plus a consensus of the three models |
| Multivariable analysis with non radiomic features (e.g.. EGFR mutation) – is expected to provide a more holistic model. Permits correlating/inferencing between radiomics and non radiomics features | +1 | 1 | All available clinical factors were included in the modeling step |
| Detect and discuss biological correlates – demonstration of phenotypic differences (possibly associated with underlying gene–protein expression patterns) deepens understanding of radiomics and biology | +1 | 0 | Not relevant to the present study |
| Cut-off analyses – determine risk groups by either the median. a previously published cut-off or report a continuous risk variable. Reduces the risk of reporting overly optimistic results | +1 | 1 | We included two endpoints (median and 6 months overall survival to study results consistency) |
| Discrimination statistics – report discrimination statistics (e.g.. C-statistic. ROC curve. AUC) and their statistical significance (e.g.. p-values. confidence intervals). One can also apply resampling method (e.g.. bootstrapping. cross-validation) | +1 (if a discrimination statistic and its statistical significance are reported)+1 (if also an resampling method technique is applied) | 1 | Three metrics were used to evaluate and compare performance of the different models |
| Calibration statistics – report calibration statistics (e.g.. Calibration-in-the-large/slope. calibration plots) and their statistical significance (e.g.. p-values. confidence intervals). One can also apply resampling method (e.g.. bootstrapping. cross-validation) | +1 (if a calibration statistic and its statistical significance are reported)+1 (if also an resampling method technique is applied) | 0 |  |
| Prospective study registered in a trial database – provides the highest level of evidence supporting the clinical validity and usefulness of the radiomics biomarker | +7 (for prospective validation of a radiomics signature in an appropriate trial) | 4 | Part of the cohort used is prospectively recruited in registered trial (NCT03199599), and was used as the testing set of the models |
| Validation – the validation is performed without retraining and without adaptation of  the cut-off value, provides crucial information with regard to credible clinical performance | -5 (if validation is missing)  +2 (if validation is based on a dataset from the same institute)  +3 (if validation is based on a dataset from another institute)  +4 (if validation is based on two datasets from two distinct institutes)  +4 (if the study validates a previously published signature)  +5 (if validation is based on three or more datasets from distinct institutes) | 2 |  |
| Comparison to ‘gold standard’ – assess the extent to which the model agrees with/is superior to the current ‘gold standard’ method (e.g., TNM-staging for survival prediction). This comparison shows the added value of radiomics | +2 | 2 | Clinical factors were included in the modeling step. A comparison with TNM stage was performed. |
| Potential clinical utility – report on the current and potential application of the model in a clinical setting (e.g.. decision curve analysis) | +2 | 0 | Not relevant to the present study |
| Cost-effectiveness analysis – report on the cost-effectiveness of the clinical application (e.g.. quality adjusted life years generated) | +2 | 0 | Not relevant to the present study |
| Open science and data – make code and data publicly available. Open science facilitates knowledge transfer and reproducibility of the study | +1 (if scans are open source)  +1 (if region of interest segmentations are open source)  +1 (if code is open source)  +1 (if radiomics features are calculated on a set of representative ROIs and the calculated features + representative ROIs are open source) | 0/+3 | Data is currently not available but will be when the recruitment will be over. All images, segmentations and clinical data will be made available in TCIA following FAIR guidelines |
| Total | 36 | 16/19 |  |
